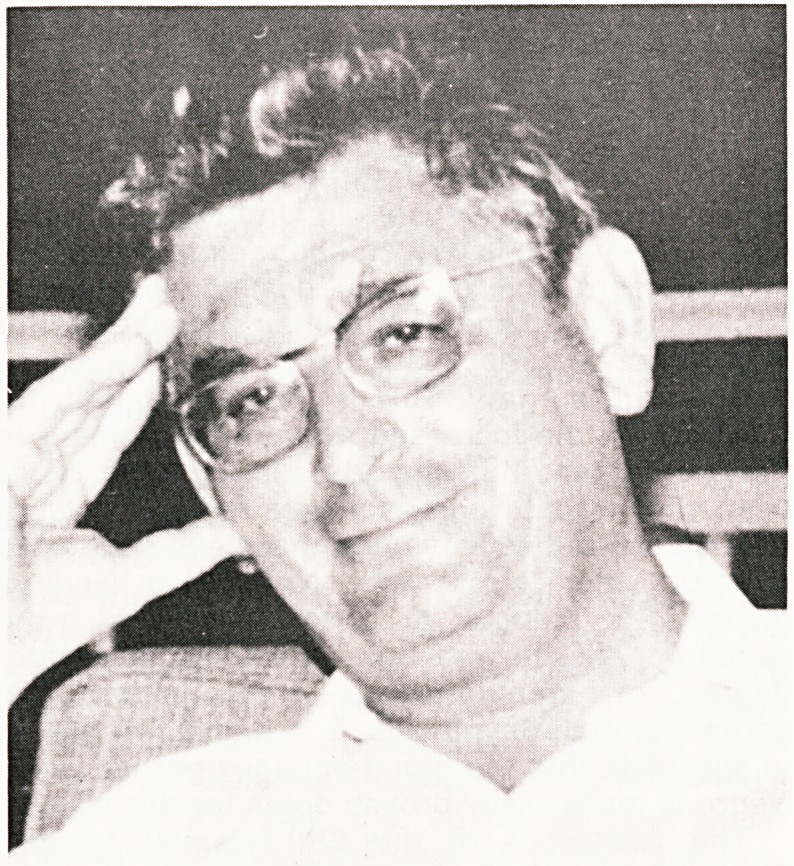# Professor Geoffrey Dixon

**Published:** 1980

**Authors:** 


### Obituaries

### Professor Geoffrey Dixon
### M.B., B.S., Ph.D.(London), F.R.C.P.Ed., F.R.C.O.G.
### 1923–80

Following a short illness, Professor H. Geoffrey Dixon, Head of the University of Bristol Department of Obstetrics and Gynaecology, died in Frenchay Hospital on 26th August 1980, at the age of 56.

Geoffrey Dixon was born in Liverpool in 1923, an only child with a non-medical family background. He received his early education at Colfe's Grammar School and after qualifying at Guy's Hospital in 1946 and completing the normal training posts in medicine and surgery, he spent two years as a Surgeon Lieutenant in the Royal Naval Volunteer Reserve. On leaving the Navy he became obstetrical house surgeon to the British Hospital for Mothers and Babies where he came under the influence and inspiration of Keith Vartan. Further training posts in obstetrics and gynaecology at the Brighton General Hospital and Hammersmith Hospital followed. Perceiving the growing importance of medicine in obstetrics and gynaecology, he chose to train as a physician rather than as a surgeon and became a member of the Royal College of Physicians of Edinburgh in 1955, subsequently being elected a Fellow in 1966. Meanwhile he had also become a member of the Royal College of Obstetricians and Gynaecologists and was elected to their Fellowship in 1964. In 1956 he was appointed Senior Lecturer and Consultant in Obstetrics and Gynaecology to Professor Dave Stewart's Department in the University of the West Indies in Jamaica. There he remained for four productive years, during which time he first worked with the then Dr. Bill Robertson on what they termed 'the placental bed'. Other contributions included work on advanced extrauterine pregnancy and on the haemoglobinopathies in relation to parturition. In 1967 he was granted a Ph.D. by the University of London for his thesis on 'Obstetrical Hypertension'. This was based on a study of the blood pressure of pregnant women in the remote rural areas of British Guiana (now Guyana). He whimsically summarised his findings as 'The nearer you approached to a mission station, the higher the women's blood pressure became'. Returning to London in 1960 he took up a similar appointment in the Institute of Obstetrics and Gynaecology at Hammersmith Hospital. During the next seven years his international reputation was established, particularly through his in-vivo studies of choriodecidual and myometrial blood flow. It was at this time that the Dixon-Robertson team was joined by a young Belgian Ph.D. student, Ivo Brosens. Together these three collaborated in studies that were to continue right up to the present time and were to shed light on the physiological vascular changes in the placental bed in normal pregnancy, and on the pathological changes in pregnancies complicated by hypertension, diabetes and fetal growth retardation. The management of maternal diabetes in pregnancy was in fact one of his long term interests, and it was a subject on which he was a widely acknowledged authority. But these were only a few of his many interests as the titles of some 75 publications and books bear witness. Included among the latter was 'Browne's Antenatal Care', the 10th and 11th editions of which he wrote with his teacher and long term friend, the late John McClure Browne of Hammersmith. He served as External Examiner to the University of the West Indies, and in the UK to the Universities of London, Manchester, Aberdeen and Belfast. He was made an honorary member of the Royal Belgian Society of Gynaecology and Obstetrics in 1967.

In 1967 Geoffrey Dixon was appointed to the Chair of Obstetrics and Gynaecology in the University of Bristol. Considerable problems confronted him on his arrival. The main difficulties were the heavy and widely spread clinical and teaching responsibilities that he inherited and the ill-matched paucity of academic staff to meet these demands. There was no quick solution. However, with a mixture of persistance and diplomacy much was achieved during the next 13 years. The academic staff was considerably expanded and with the opening of the new Bristol Maternity Hospital in 1975, to which the main academic unit moved, the clinical and teaching duties of the Department became more centralised. At the same time plans were laid for the re-organisation of the undergraduate teaching of maternity and newborn care utilising the resources of the whole South West Region as well as the teaching hospitals. In collaboration with senior members of his own Department a manual on 'Undergraduate Obstetrics and Gynaecology' was prepared which is shortly to be published by John Wright and Sons of Bristol. Important as these achievements were, perhaps his greatest contribution to maternity care in Bristol was in his perinatal approach to obstetric problems. Not for him the narrow territorial approach as to whether the newborn baby belonged to the obstetrician or the paediatrician. To him the baby belonged to the mother and he welcomed any help that would ensure that the child went home healthy and fit. Paediatricians were made welcome on his antenatal ward rounds, given tremendous support, and consulted on problems of mutual interest. As a senior paediatric colleague exclaimed on hearing of his death, 'Paediatrics and the newborn have lost a very good friend'.

Geoffrey Dixon was not an easy man to get to know and to those who did not know him he sometimes appeared difficult. He also had a disconcerting habit of, on occasion, being outrageously rude in public – but only to colleagues that he knew and liked sufficiently to be sure that they would realise that this was his curiously idiosyncratic way of expressing appreciation for their help. Like many sensitive people he cloaked his true nature with a somewhat hard outer crust. Those that were fortunate enough to penetrate this shell and his shyness and be admitted to his friendship knew him as a genial companion, a generous host, and a devotee of Monty Python. He loved good food and was as happy in the kitchen preparing it as eating it afterwards with his wife and friends. His other great spare-time interests were underwater swimming and fishing in tropical waters, hobbies dating back to his years in Jamaica. Indeed, he used to say that his greatest claim to fame was being fifth author of a paper by his great friend Tom Goreau, and others, on the discovery of a new coral in the Caribbean. Perhaps though, the most outstanding of all his personal qualities was the consideration and unstinting loyalty that he gave to his friends and to the members of his Department. His junior staff grew to appreciate that if they had served him well, he would not spare in his efforts to ensure that they obtained good posts further up the ladder. His students too, appreciated him and awarded him their ultimate accolade, an MCP tie! The widespread esteem and affection in which he was held is best summed up in a letter the writer received from Professor Dixon's long-time personal secretary, Joan Davies. She wrote, 'This week I have been inundated with visits and phone calls from all sorts of people asking if there was anything they could do to help – porters, domestics, medical, nursing and administrative staff. The Bristol Maternity Hospital has been completely shattered and we all feel that we have lost not only the Head of the Department but a friend. I could not have worked for a better boss. He was kind, considerate and most helpful, especially if any of us was in any kind of personal difficulty and distress. He was a most affectionate man who gave of his best to his staff and was a staunch ally. This goes not only for me, who perhaps knew him as well as anyone, but for all of us in the Department'.

The funeral service on Friday, 29th August was held at the quiet and beautiful church of St. Mary the Virgin in Leigh Woods. Friends from home and abroad gathered to share their grief with that of his adored daughters and his very dearly loved wife Gillian. Our sympathy particularly goes out to Gillian Turner Dixon; as senior member of the University Department she has now taken over as a temporary head of Obstetrics and Gynaecology.

## Figures and Tables

**Figure f1:**